# While end-of-production sole-source lighting at a moderate intensity increased nutrient, water-soluble vitamin, and carotenoid content, the anthocyanin concentration of red leaf lettuce decreased

**DOI:** 10.3389/fpls.2026.1699278

**Published:** 2026-02-19

**Authors:** Devin Brewer, Kellie J. Walters, Jennifer K. Boldt, Roberto G. Lopez

**Affiliations:** 1 Department of Horticulture, Michigan State University, East Lansing, MI, United States; 2 Department of Plant Sciences, University of Tennessee, Knoxville, TN, United States; 3 United States Department of Agriculture - Agricultural Research Service Application Technology Research Unit, Toledo, OH, United States

**Keywords:** blue light, coloration, controlled-environment agriculture, leafy greens, lettuce, light quality, red light, water-soluble vitamins

## Abstract

In controlled environments, light intensity and quality can be manipulated to enhance beneficial phytochemicals, vitamins, and foliage color by increasing anthocyanin levels before harvest. Our objectives were to quantify whether a 50% reduction in end-of-production (EOP) light intensity, enriched with blue (B) light alone or with red (R) light, would yield red leaf lettuce of comparable or higher quality than that grown under high-intensity white light. Seedlings of red leaf lettuce (*Lactuca sativa*) ‘Barlach’, ‘Rouxai’, and ‘Thurinus’ were grown under light-emitting diodes (LEDs) that provided a photosynthetic photon flux density (PPFD) of 300 µmol·m^−2^·s^−1^ with a ratio (%) of 19:39:39:3 B:green:R:far-red light. During the last 6–8 days of production, plants were either left in the same conditions or placed under LEDs providing a ratio (%) of 100:00, 75:25, or 50:50 B:R light and a PPFD of 150 µmol·m^−2^·s^−1^. Compared to untreated plants, the shoot fresh mass of ‘Rouxai’ was 16%, 17%, and 21% lower, and the shoot dry mass was 25%, 27%, and 29% lower under 100:00, 75:25, and 50:50 B:R EOP treatments, respectively. ‘Thurinus’ exposed to 100:00 B:R EOP lighting contained 22%, 22%, 47%, 35%, 24%, 10%, 40%, 31%, 25%, 50%, and 38% greater N, P, Ca, Mg, S, B, Cu, Fe, Mn, Mo, and Zn concentrations, respectively, compared to the control. Plants exposed to EOP generally developed increased concentrations of water-soluble vitamins. Vitamin C of ‘Barlach’, ‘Rouxai’, and ‘Thurinus’ was between 37% and 42%, 36% and 45%, and 49% and 57% greater, respectively, when exposed to EOP B or B:R sole-source lighting. The carotenoid concentration of ‘Rouxai’ under 100:00 B:R was 29% greater than that of the control. Each cultivar developed the highest concentration of violaxanthin, neoxanthin, zeaxanthin, and α-carotene when not exposed to EOP lighting. Under EOP lighting, ‘Barlach’, ‘Rouxai’, and ‘Thurinus’ accumulated between 52% and 68%, 55% and 57%, and 33% and 43% lower anthocyanin concentrations, respectively, than the control plants. Our results indicate that maintaining a higher light intensity during the EOP phase was more effective at increasing anthocyanin production in red leaf lettuce than lowering light intensity and increasing the fraction of blue light.

## 1 Introduction

Consumer demand for fresh, local, pesticide-free, and year-round leafy greens has spurred the growth of greenhouse and indoor controlled-environment (CE) production. In the United States, from 2014 to 2019, the total sales of lettuce (*Lactuca sativa* L.) produced in CEs increased by 28% from $56 to $71 million (USDA, 2015, 2020). The increased demand for lettuce, especially red leaf varieties, is primarily driven by their health properties, which are attributed to their high fiber content, phenolic compounds such as anthocyanins, and carotenoids (Llorach et al., 2008; Nicolle et al., 2004).

Light quality refers to the various wavelengths of the light spectrum, which correlate to different colors and categories of light. Sunlight delivers a spectrum including visible, ultraviolet (UV), and infrared light. However, plants primarily utilize photosynthetically active radiation (PAR), which includes blue (B, 400–500 nm), green (G, 500–600 nm), and red (R, 600–700 nm), for photosynthesis (Runkle, 2006). Additionally, they perceive and are affected by UV-B (280–315 nm), UV-A (315–400 nm), and far-red (FR; 700–750 nm) light (Kohler, 2020). Furthermore, each waveband is associated with unique physiological and morphological effects that can vary among plant species and cultivars (Bantis et al., 2018; Samuolienė et al., 2013).

Within CEs, photosynthesis, growth, yield, and quality can be maximized by customizing environmental parameters, such as light quality. Furthermore, research has found that growth similar to that achieved under sunlight can be achieved using combinations of primarily B and R light (Bian et al., 2018; Viršilė et al., 2020). As a result, many commercial horticultural light-emitting diode (LED) fixtures emit B and R light primarily due to their high photosynthetic efficiency and photon efficacy (Nelson and Bugbee, 2014; Park and Runkle, 2018).

The presence of anthocyanins provides a diversity of foliage and flower colors, including red, blue, and purple pigmentation in microgreens, basil (*Ocimum basilicum*), kale (*Brassica oleracea* var. *sabellica*), and lettuce, a metric that consumers consider when purchasing produce (Goins et al., 1998; Li and Kubota, 2009; Samuoliené et al., 2012). Anthocyanins serve many roles in plants, including as protective pigments to counteract oxidative damage. Synthesis may be induced by wounding, pathogen infection, exposure to excessive photon flux densities, UV radiation, and/or moderate B or B:R light, low temperature, and phosphorus nutrient deficiency (Boldt et al., 2014; Owen and Lopez, 2015). However, anthocyanin production may be inhibited by environmental conditions, such as low light intensity and high mean daily temperature (MDT) (Kleinhenz et al., 2003).

Carotenoids are a class of yellow, orange, and red pigments within plants that contribute to coloration in many fruits and vegetables (Edge et al., 1997). Similar to anthocyanins, these compounds provide valuable health benefits, such as reducing the risk of age-related macular degeneration, cataracts, and certain types of cancer (Kaulmann and Bohn, 2014). Carotenoid accumulation can be influenced by light intensity and quality, temperature, and nutrient availability. Among environmental factors that induce the accumulation of anthocyanins and carotenoids, light quality and intensity can be readily altered in CEs with adjustable-intensity LED fixtures that provide different light qualities (Viršilė et al., 2020).

Water-soluble vitamins (WSVs) include the B vitamin complex and ascorbic acid (vitamin C), both micronutrients required by all life forms to sustain growth and development (Liu et al., 2022). Thiamine (B_1_), riboflavin (B_2_), niacin (B_3_), pantothenic acid (B_5_), pyridoxine (B_6_), biotin (B_7_), and folic acid (B_9_) make up this B vitamin complex. Plants synthesize these compounds as needed; however, they may fail to do so under environmental stressors (Hanson et al., 2016). Currently, research on the impact of environmental parameters on WSV accumulation is limited mainly to vitamin C, with little to no research focused on the B vitamin complex (Hanson et al., 2016).

Numerous studies have investigated the effects of sole-source light intensity and quality on the growth, nutritional content, and yield of lettuce and other leafy greens. However, less attention has been given to end-of-production (EOP) lighting treatments. EOP supplemental and sole-source lighting are novel strategies in which environmental parameters, such as light intensity and/or quality, are changed near the end of the cropping cycle, inducing stress responses that enhance desired characteristics, including biomass, coloration, mineral nutrition, carotenoid, and anthocyanin accumulation (Gómez and Jiménez, 2020; Kelly and Runkle, 2023; Zhu et al., 2024). For instance, exposing greenhouse- and indoor-grown lettuce to short-duration EOP supplemental light at different intensities of UV-A, B, and/or R can improve the coloration of red leaf lettuce (Gómez and Jiménez, 2020; Owen and Lopez, 2015).

Currently, there is a lack of research on the effectiveness of EOP sole-source lighting providing ratios of B and R light to improve anthocyanin, carotenoid, WSV, and mineral nutrient content, and the extent to which EOP treatments may affect production costs for indoor farms. The majority of previous studies have indicated that high B light intensities are required for anthocyanin synthesis (Owen and Lopez, 2015; Li et al., 2021; Page et al., 2012; Zhang et al., 2018). However, Zhu et al. (2024) recently reported that anthocyanin production depends on both B light intensity and application duration during a 24-h period. For instance, providing 4 h of 270 µmol·m^−2^·s^−1^ of EOP B light for 7 days was less effective than providing 135 µmol·m^−2^·s^−1^ of EOP B light for 8 h. Additionally, they found that a high-intensity B light of 270 µmol·m^−2^·s^−1^ applied over a short duration was more effective at enhancing chlorophylls and carotenoids, while low-intensity B light over a long duration resulted in the highest yield (Zhu et al., 2024).

Therefore, the objective of this study was to produce red leaf lettuce under the environmental conditions that Tarr et al. (2023) reported to maximize yield and to quantify the influence of sole-source EOP lighting providing a lower light intensity of B or different ratios of B + R light on anthocyanin, carotenoid, WSV, and mineral nutritional concentration as well as coloration, plant quality, and yield of red leaf lettuce. We hypothesized that reducing EOP sole-source lighting to 150 µmol·m^−2^·s^−1^ would increase lettuce anthocyanin, carotenoid, WSV, and mineral nutrient concentrations, given the lack of background solar radiation, without sacrificing yield and reducing energy costs. Furthermore, we hypothesized that the combination of B:R light would be more effective at enhancing anthocyanin concentration and coloration than monochromatic B light alone.

## 2 Materials and methods

### 2.1 Plant material and propagation conditions

Seeds of red oakleaf ‘Rouxai’, Salanova butterhead ‘Barlach’, and cos lettuce ‘Thurinus’ (Rijk Zwaan USA, Salinas, CA, USDA) were sown into 200-cell (2.5 cm × 2.5 cm) rockwool plugs (AO 25/40 Starter Plugs, Gordan, Milton, ON, Canada) based on the methods outlined by Tarr et al. (2023) and detailed below. The varieties were chosen based on their suitability for hydroponic growth and resistance to the physiological disorder, tip burn. Rockwool plugs (AO 25/40 Starter Plugs, Gordan, Milton, ON, Canada) were presoaked in deionized (DI) water with a pH of 4.4 to 4.5 for 30 minutes. The water pH was adjusted using diluted (1:31) 95%–98% sulfuric acid (J.Y. Baker, Inc., Phillipsburg, NJ, USA). Trays were then covered with translucent plastic domes for 3 days to maintain high humidity during germination. Trays were then placed in one of three walk-in growth chambers (Hotpack environmental room UWP 2614-3; SP Scientific, Warminster, PA, USA) with an MDT of 22°C, CO_2_ concentration of 500 μmol·mol^−1^, relative humidity (RH) of 60%, and a vapor-pressure deficit (VPD) of 1.1 kPa. LED fixtures (GreenPower LED production module 3.0; Philips, Amsterdam, Netherlands) provided a photosynthetic photon flux density (PPFD) of 180 μmol·m^−2^·s^−1^ and a light ratio (%) of 19:39:39:3 B:G:R:FR for 24 h. After 3 days, the photoperiod was reduced to 20 h·day^−1^ until seedlings were transplanted on day 11. Seedlings were sub-irrigated with deionized water supplemented with water-soluble fertilizer, providing the following (in mg·L^−1^): 125 N, 18 P, 138 K, 73 Ca, 47 Mg, 1.56 Fe, 0.52 Mn, 0.36 Zn, 0.21 B, 0.21 Cu, 35 S, and 0.01 Mo (12N–4P–16K RO Hydro FeED; JR Peters, Inc., Allentown, PA, USA). The pH and electrical conductivity (EC) of the solution were adjusted to 5.6 and 1.6 mS·cm^−1^, respectively, using a pH/EC probe (HI 991301 pH/TDS/Temperature Monitor; Hanna Instruments, Smithfield, RI, USA).

### 2.2 Hydroponic systems

On day 11 of each replication, 36 seedlings of each cultivar were transplanted 20 cm apart into three 250-L, 0.9-m-wide by 1.8-m-long deep-flow hydroponic systems (Active Aqua premium high-rise flood table; Hydrofarm, Petaluma, CA, USA) with one tank in each walk-in growth chamber (Hotpack environmental room UWP 2614-3; SP Scientific). Each hydroponic tank was covered with a floating 4-cm-thick extruded polystyrene foam sheet (R-3 Square Edge Rigid Foam Board Insulation Sheathing, Owens Corning, Toledo, OH, USA). The foam sheets had 4-cm-diameter holes drilled in them to accommodate plastic net baskets that held rockwool plugs and seedlings in contact with the nutrient solution. DI water was supplemented with water-soluble fertilizer, providing the following (mg·L^−1^): 150 N, 22 P, 166 K, 87 Ca, 25 Mg, 1.9 Fe, 0.62 Mn, 0.44 Zn, 0.25 B, 0.25 Cu, and 0.01 Mo (Jack’s 12N–4P–16K; JR Peters, Inc.), and 0.31 g·L^–1^ magnesium sulfate (Pennington Epsom Salt, Madison, GA, USA). The pH and EC were measured and adjusted daily to maintain an EC of 1.7 ± 0.05 mS·cm^−1^ by adding DI water or concentrated nutrient solution as needed, while the pH was adjusted to 5.6 ± 0.05 using potassium bicarbonate and sulfuric acid. A dissolved oxygen concentration of 9.8 ± 0.03 mg·L^−1^ was maintained using air pumps (Active Aqua 70 L·min^−1^ commercial air pump; Hydrofarm) connected to air stones (Active Aqua air stone round 10.2 cm × 2.5 cm; Hydrofarm).

### 2.3 Growth chamber environmental conditions

Following the protocol of Tarr et al. (2023), the air day/night temperature (17/7 h) 28°C/21°C (MDT 26°C) was measured every 5 seconds using a resistance temperature detector (Platinum RTD RBBJL-GW05A-00-M 36B; SensorTec, Inc., Fort Wayne, IN, USA) and logged by a C6 controller (Environmental Growth Chambers, Chagrin Falls, OH, USA). A light intensity of 300 μmol·m^−2^·s^−1^ was provided for 17 h·day^−1^ by LED fixtures (GreenPower LED production module 3.0; Phillips, Amsterdam, Netherlands), achieving a daily light integral of 18.4 mol·m^−2^·day^−1^ with the LED fixtures mounted 10–12 cm above the crop canopy. Water and canopy temperature and PPFD were monitored using a thermistor (ST-100; Apogee Instruments, Logan, UT, USA), infrared thermocouple (OS36-01-T-80F; Omega Engineering, Inc., Norwalk, CT, USA), and quantum sensor (LI-190R; LI-COR Biosciences, Lincoln, NE, USA), respectively, every 30 seconds, and means were logged each hour by a CR-1000 datalogger (Campbell Scientific, Logan, UT, USA). The CO_2_ concentration was monitored and maintained using a CO_2_ sensor (GM86P; Vaisala, Helsinki, Finland) at 800 μmol·mol^−1^ during the day with compressed CO_2_ injection and logged by a C6 Controller (Environmental Growth Chambers) every 5 seconds. A VPD of 1.03 kPa was maintained via day and night RH set points of 70% and 55%, respectively (**Table 1**). Overhead fans were used to maintain an average air velocity of 0.7 m^−3^·s^−1^ at plant height, measured at transplant using an anemometer (LM-8000A; Lutron, Coopersburg, PA, USA).

**Table 1:** The photosynthetic photon flux density (PPFD) during production (Prod.) and end-of-production (EOP) sole-source lighting providing a blue (B) and red (R) light ratio (%); mean (± SD) day and night air, canopy, and nutrient solution temperature; and carbon dioxide (CO_2_) concentration and vapor-pressure deficit (VPD) during 30 days of indoor deep-flow hydroponic production of red leaf lettuce (*Lactuca sativa*) ‘Barlach’, ‘Rouxai’, and ‘Thurinus’.

Rep.	EOP (B:R)	Prod. PPFD (µmol·m^−2^·s^−1^)	EOP PPFD (µmol·m^−2^·s^−1^)	Temperature (°C)				CO_2_ (μmol·mol^−1^)	VPD (kPa)
				Air day	Air night	Canopy	Nutrient		
1	100:00	297.8 ± 4.7	149.7 ± 13.0	26.9 ± 0.3	22.8 ± 0.4	26.5 ± 3.2	25.6 ± 3.2	790.7 ± 84.2	1.02 ± 0.09
	75:25	293.2 ± 5.1	152.3 ± 13.4	27.3 ± 0.1	22.0 ± 0.4	28.7 ± 2.9	25.6 ± 1.3	791.7 ± 82.0	1.04 ± 0.10
	50:50	296.8 ± 8.2	154.8 ± 10.7	27.4 ± 0.1	22.9 ± 0.4	27.9 ± 2.9	25.9 ± 1.1	782.5 ± 90.4	1.00 ± 0.13
	Control	300.2 ± 7.8	301.4 ± 4.5	27.3 ± 0.2	22.1 ± 0.3	27.8 ± 2.0	25.5 ± 1.6	795.2 ± 82.4	1.02 ± 0.05
2	100:00	292.5 ± 12.5	148.6 ± 17.4	26.9 ± 0.4	22.4 ± 0.4	26.0 ± 3.1	25.8 ± 2.9	806.1 ± 45.0	1.07 ± 0.07
	75:25	293.2 ± 19.8	153.8 ± 4.5	27.6 ± 0.3	22.5 ± 0.4	28.7 ± 3.0	25.6 ± 1.4	805.8 ± 39.8	1.06 ± 0.17
	50:50	294.9 ± 17.8	150.0 ± 8.9	27.6 ± 0.1	22.4 ± 0.4	27.9 ± 2.9	25.9 ± 1.2	796.3 ± 42.9	1.04 ± 0.11
	Control	294.5 ± 13.2	298.5 ± 3.9	27.3 ± 0.9	22.3 ± 0.1	27.5 ± 3.1	26.0 ± 1.3	801.4 ± 42.1	1.05 ± 0.03
3	100:00	296.4 ± 17.1	152.7 ± 13.5	28.1 ± 0.6	21.7 ± 0.3	28.0 ± 3.3	25.3 ± 1.6	804.9 ± 22.7	1.05 ± 0.08
	75:25	292.3 ± 16.2	155.3 ± 5.6	27.8 ± 0.1	22.0 ± 0.4	28.3 ± 2.8	25.9 ± 1.2	796.5 ± 24.7	1.01 ± 0.15
	50:50	297.8 ± 4.7	154.1 ± 4.8	27.1 ± 0.4	21.9 ± 0.4	26.4 ± 3.2	25.4 ± 3.3	806.4 ± 18.0	1.01 ± 0.10
	Control	302.7 ± 10.1	300.5 ± 2.4	28.3 ± 0.5	22.2 ± 0.2	27.5 ± 2.8	26.0 ± 2.3	794.3 ± 28.1	1.08 ± 0.09

Twenty-four days after transplant in the hydroponic system, ‘Thurinus’ was placed under LEDs providing 150 μmol·m^−2^·s^−1^ PPFD (GreenPower LED production module 3.0; Philips, Amsterdam, Netherlands) of a ratio (%) of 100:0, 75:25, or 50:50 B:R light for 6 days (**Table 1**). This process was repeated on day 30 for 8 and 6 days for ‘Barlach’ and ‘Rouxai’, respectively, as these cultivars required more time to reach marketability and/or required a longer duration to produce anthocyanins (Brewer et al., 2024). All other environmental conditions remained unchanged. Plants placed under the control treatment were not exposed to EOP treatments, receiving the same light intensity (300 μmol·m^−2^·s^−1^) and quality until harvest.

### 2.4 Data collection and postharvest analysis

Foliage coloration measurements were conducted using a tristimulus colorimeter (Chroma Meter CR-400; Konica Minolta Sensing, Inc., Chiyoda, Japan) on 13 plants of ‘Rouxai’, ‘Barlach’, and ‘Thurinus’ from each treatment on days 24, 26, 28, and 30 for ‘Thurinus’; days 30, 32, 34, and 36 for ‘Rouxai’; and days 30, 32, 34, 36, and 38 for ‘Barlach’, as described by Owen and Lopez (2015). L* measured leaf lightness. Chlorophyll fluorescence was measured on the three most recent fully expanded leaves of each plant. After dark acclimation for >15 minutes using manufacturer-supplied clips, each leaf was exposed to 3,500 µmol·m^–2^·s^–1^ of R light (peak wavelength 650 nm) to achieve saturation of photosystem II. Fluorescence was measured, averaged, and reported as F_v_/F_m_ using a portable chlorophyll fluorescence meter (Handy Plant Efficiency Analyzer; Hansatech Instruments Ltd., Norfolk, UK).

‘Thurinus’, ‘Rouxai’, and ‘Barlach’ were harvested 30, 36, and 38 days after sowing, respectively. Shoot fresh mass (SFM) and shoot dry mass (SDM; g), the length and width (cm) of the sixth most recent fully expanded leaf, and leaf number (when >5 cm) were measured on 13 plants of each cultivar per treatment. Plant height from the basal leaves to the apical meristem, plant width at the widest point (dia_1_), and perpendicular from the widest point (dia_2_), as well as the presence of tip burn, were recorded. To provide an integrated measurement of plant size, the growth index (GI) equation $\frac{\frac{\left(\text{d}\dot{\text{i}}{\text{a}}_{1}+\text{d}\text{i}{\text{a}}_{2}\right)}{2}+\mathrm{ht}}{2}\;$ (Krug et al., 2010) was used. After obtaining the SFM of each plant, the remaining leaves were separated to obtain approximately 40 g of biomass. The biomass was placed into a polyethylene sampling bag and flash-frozen by submersion in liquid N. Frozen samples were stored at –80°C until freeze-dried. The remaining plant material from each sample was placed in a forced-air drier maintained at 75°C for at least 3 days. Freeze- and oven-dried samples were then weighed, and the weights were added and recorded.

Mineral nutrient analysis was conducted on oven-dried samples from 13 plants of ‘Rouxai’ and ‘Thurinus’, and treatment according to the methods described by Brewer et al. (2024).

Thirteen freeze-dried tissue samples were homogenized using a ceramic mortar and pestle containing liquid N. Total anthocyanin extraction and quantification were conducted according to a method, as reported by Darby et al., 2024. The carotenoids α-carotene, β-carotene, lutein, neoxanthin, violaxanthin, and zeaxanthin, as well as chlorophylls *a* and *b*, were extracted and quantified according to the method described by Darby et al., 2024

Water-soluble vitamins C, B_1_, B_3_, and B_6_ were extracted using a method and reported by Darby et al., 2024.

### 2.5 Data analysis

The experiment was arranged in a randomized split-block design, with each growth chamber containing 13 plants of each cultivar and serving as a block. Each EOP treatment was randomly assigned to a chamber across the repetitions. The experiment was completed thrice in time, and the growth chamber lighting treatments were randomized. Data were analyzed separately by cultivar using SAS (version 9.4; SAS Institute, Cary, NC, USA) mixed model procedure (PROC MIXED) for analysis of variance (ANOVA), and pairwise comparisons were performed using the Tukey–Kramer difference test (p ≤ 0.05).

## 3 Results

### 3.1 Shoot fresh and dry mass

Compared to those of the untreated control, the SFM and SDM of ‘Rouxai’ and ‘Thurinus’ were reduced when exposed to 6 days of B:R EOP sole-source lighting (**Table 2**). For example, the SFM of ‘Rouxai’ was 16% (28.7 g), 17% (29.4 g), and 21% (34.6 g) lower under the 100:00, 75:25, and 50:50 B:R EOP treatments, respectively, than that of the control. Similarly, the SDM of ‘Rouxai’ placed under 100:00, 75:25, and 50:50 B:R EOP treatments was 25% (1.6 g), 27% (1.7 g), and 29% (1.9 g) lower than that of the control, respectively (**Table 2**). Placing ‘Thurinus’ under 75:25 and 50:50 B:R EOP treatments resulted in a 30% (43.0 g) and 27% (1.5 g) decrease in SFM and a 28% (42.7 g) and 25% (1.4 g) decrease in SDM, respectively (**Table 2**). However, the SFM and SDM of ‘Barlach’ were not altered by any EOP treatment (**Table 2**).

**Table 2:** Influence of end-of-production (EOP) sole-source lighting providing a ratio (%) of 100:00, 75:25, and 50:50 blue:red (B:R) light or no EOP treatment (control) on leaf number (no.), shoot fresh and dry mass, growth index, chlorophyll fluorescence (F_v_/F_m_), and total chlorophyll content of red leaf lettuce (*Lactuca sativa*) ‘Barlach’, ‘Rouxai’, and ‘Thurinus’.

EOP (B:R)	Leaf (no.)	Fresh mass (g)	Dry mass (g)	Growth index	Chlorophyll fluorescence (F_v_/F_m_)	Total chlorophyll (µg-mL^−1^)
‘Barlach’						
Control	60.6 a	180.4 a	6.3 a	18.4 b	0.8391 a	5.1 a
100:00	56.7 b	173.4 a	5.9 a	21.2 a	0.8364 a	9.0 b
75:25	58.9 ab	171.7 a	5.8 a	19.8 b	0.8468 a	5.0 a
50:50	60.7 a	170.7 a	5.8 a	19.3 b	0.8381 a	5.8 a
‘Rouxai’						
Control	28.1 ab	178.7 a	6.4 a	21.6 a	0.8351 a	4.8 b
100:00	23.0 c	150.0 b	4.8 b	21.7 a	0.8403 a	8.6 a
75:25	26.3 b	149.3 b	4.7 b	20.8 a	0.8429 a	4.9 b
50:50	29.9 a	144.1 b	4.5 b	20.6 a	0.8414 a	3.8 b
‘Thurinus’						
Control	24.9 a	142.9 a	5.6 a	26.1 b	0.8281 a	6.0 b
100:00	19.8 b	116.3 ab	4.6 ab	28.2 a	0.8363 a	16.3 a
75:25	21.3 b	99.9 b	4.2 b	25.0 b	0.8373 a	8.9 b
50:50	24.6 a	100.2 b	4.3 b	26.0 b	0.8349 a	11.3 b

Data represent the mean of three replications and cultivars with 13 samples. Different letters within columns indicate significantly different means according to Tukey’s honestly significant difference test (p < 0.05).

### 3.2 Plant morphology and pigmentation

Leaf number was generally lower as the EOP B light percentage increased. For instance, ‘Barlach’ and ‘Rouxai’ placed under the 100:00 B:R EOP treatment unfolded four and seven fewer leaves, respectively, compared to plants under the 50:50 B:R treatment, and four and five fewer leaves compared to the control (**Table 2**). Despite this, the GI of ‘Barlach’ was the greatest under the 100:00 B:R EOP treatment and 13% greater than that of the control. However, the GI of all cultivars was similar to that of the control when placed under the 75:25 or 50:50 B:R EOP treatments (**Table 2**). No incidence of tip burn was observed. Chlorophyll fluorescence was not influenced by any EOP treatment. However, the total chlorophyll content of all three cultivars was higher under the 100:00 B:R EOP treatment compared to the other treatments (**Table 2**).

**Figure 1:**
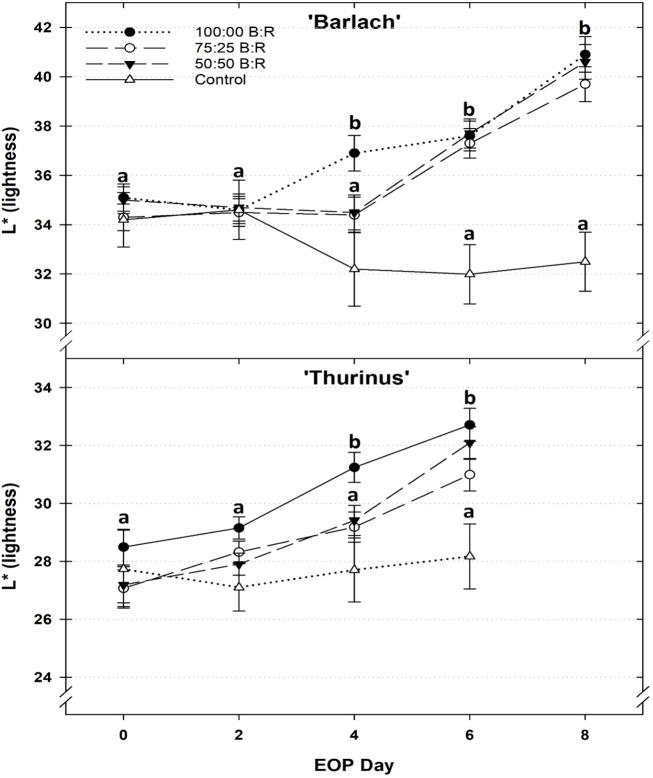
Effect of end-of-production (EOP) sole-source lighting providing a ratio (%) of 100:00, 75:25, and 50:50 blue:red (B:R) light or no EOP treatment (control) on L* of red leaf lettuce (*Lactuca sativa*) ‘Barlach’ on days 0, 2, 4, 6, and 8, and of ‘Thurinus on days 0, 2, 4, and 6. Different letters within each cultivar are significantly different based on Tukey’s honestly significant difference test (p < 0.05). Error bars show standard error.

EOP treatment and duration influenced the foliage lightness (L*) of ‘Barlach’ and ‘Thurinus’ (**Figure 1**), but not that of ‘Rouxai’. When exposed to the 100:00 B:R EOP treatment for 4 days, the L* of ‘Barlach’ was 13% greater than that of the control, indicating a lighter color than both the pretreatment measurement and the control. On day 6, the L* of ‘Barlach’ exposed to the 100:00, 75:25, and 50:50 B:R EOP treatments was 15%, 14%, and 15% greater, respectively, than that of the control. By day 8, the difference in L* between ‘Barlach’ exposed to the 100:00, 75:25, and 50:50 B:R EOP treatments and control treatment increased to 18%, 20%, and 20%, respectively (**Figure 1**). On day 4, the L* of ‘Thurinus’ exposed to the 100:00 B:R EOP treatment was 7% greater than that of the control. By day 6, the L* of ‘Thurinus’ under the 100:00, 75:25, and 50:50 B:R EOP treatments was 13%, 9%, and 12% greater, respectively, than that of the control (**Figure 1**).

### 3.3 Mineral nutrient concentration

**Figure 2:**
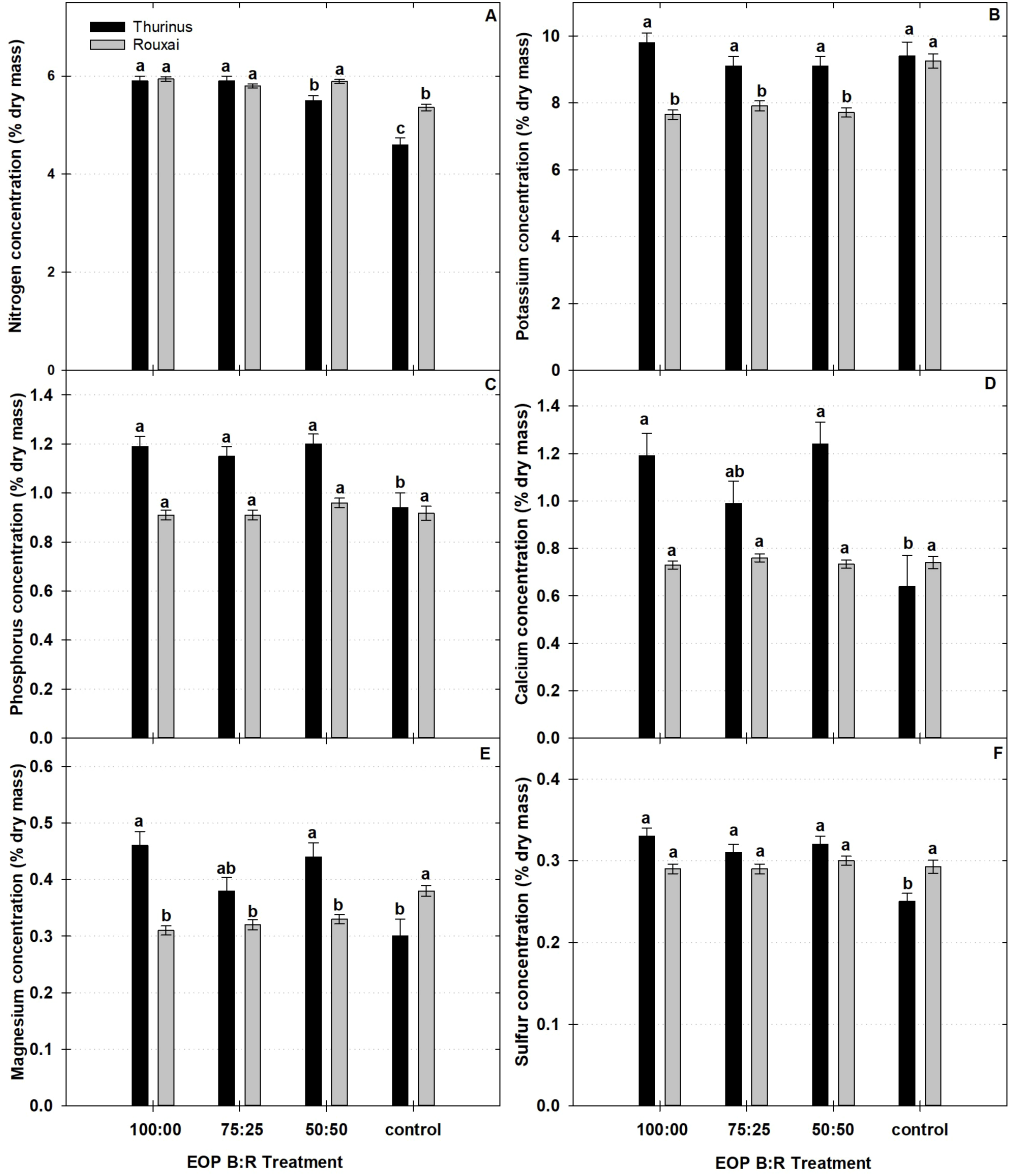
Concentrations of macronutrients {**(A)** nitrogen, **(B)** potassium, **(C)** phosphorus, **(D)** magnesium, **(E)** calcium, and **(F)** sulfur] in leaf tissues of red leaf lettuce (*Rouxai sativa*) ‘Barlach’ and ‘Thurinus’ under end-of-production (EOP) sole-source lighting providing a ratio (%) of 100:00, 75:25, and 50:50 blue:red (B:R) light or no EOP treatment (control). Different letters within each cultivar are significantly different based on Tukey’s honestly significant difference test (p < 0.05). Error bars represent the standard error of the mean.

**Figure 3:**
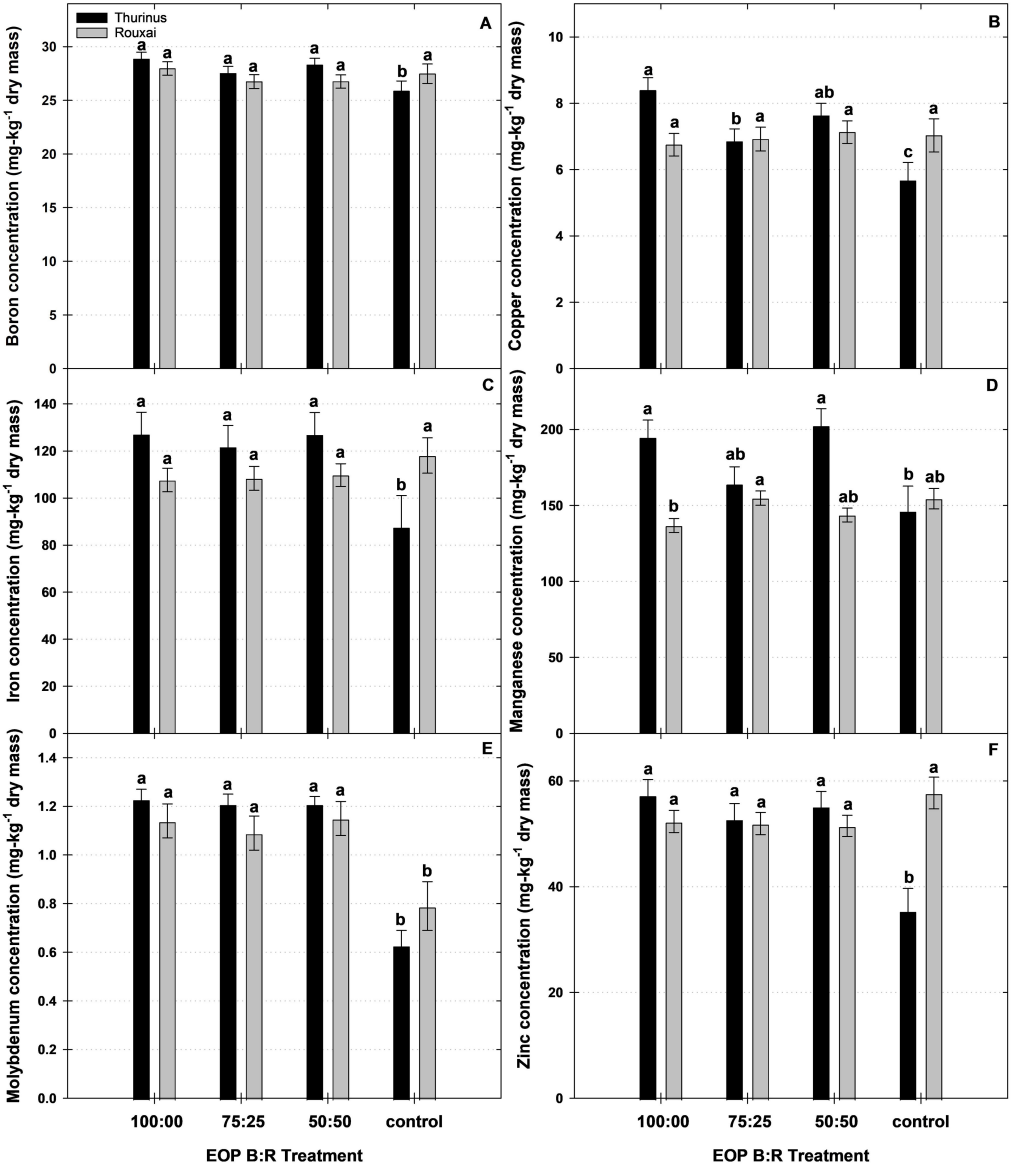
Concentrations of micronutrients [**(A)** boron, **(B)** copper, **(C)** iron, **(D)** manganese, **(E)** molybdenum, and **(F)** zinc] in leaf tissues of red leaf lettuce (*Lactuca sativa*) ‘Barlach’ and ‘Thurinus’ under end-of-production (EOP) sole-source lighting providing a ratio (%) of 100:00, 75:25, and 50:50 blue:red (B:R) light or no EOP treatment (control). Different letters within each cultivar are significantly different based on Tukey’s honestly significant difference test (p < 0.05). Error bars represent the standard error of the mean.

While a few differences in ‘Rouxai’ and ‘Thurinus’ occurred between the three B-enriched EOP treatments, macronutrient and micronutrient concentrations often differed relative to the higher-light control (**Figures 2**, **3**). For instance, ‘Rouxai’ exposure to 100:00 B:R possessed 10% and 28% greater nitrogen (N) and molybdenum (Mo), respectively, and 17% and 18% lower potassium (K) and magnesium (Mg) concentrations than the control, respectively. Under the 75:25 B:R EOP sole-source lighting treatment, ‘Rouxai’ had 7% and 27% greater N and Mo, and 14% lower K and Mg concentrations, respectively, than the control. Lastly, when ‘Rouxai’ was exposed to the 50:50 B:R EOP treatment, the concentrations of N and Mo were 8% and 30% higher than those of the control, respectively, while the concentrations of K and Mg were 16% and 11% lower than those of the control, respectively.

Compared to untreated plants, ‘Thurinus’ exposed to 100:00 B:R EOP lighting contained 22%, 22%, 47%, 35%, 24%, 10%, 40%, 31%, 25%, 50%, and 38% greater N, P, calcium (Ca), Mg, sulfur (S), boron (B), copper (Cu), iron (Fe), manganese (Mn), Mo, and zinc (Zn) concentrations, respectively. Similarly, under the 75:25 B:R EOP treatment, the concentrations of N, P, S, B, Cu, Fe, Mo, and Zn were 22%, 18%, 19%, 7%, 18%, 28%, 47%, and 33% greater, respectively, than those of the control. ‘Thurinus’ exposed to the 50:50 B:R EOP treatments contained 16%, 22%, 48%, 32%, 22%, 9%, 25%, 28%, 47%, and 36% greater N, P, Ca, Mg, S, B, Cu, Fe, Mn, Mo, and Zn concentrations, respectively, compared to the control (**Figures 2**, **3**).

### 3.4 Water-soluble vitamins

Exposure to EOP lighting treatment generally increased WSV concentration across all three cultivars. ‘Barlach’ exposed to the 50:50 B:R EOP treatment contained a 68% greater concentration of vitamin B_1_ than the control. Additionally, ‘Barlach’ placed under each EOP treatment showed a higher vitamin C concentration than the control, ranging from 40% to 43% (**Table 3**). ‘Rouxai’ exposed to the 100:00, 75:25, and 50:50 B:R EOP treatments displayed 60%, 65%, and 60% greater concentrations of vitamin B_3_, respectively, than the control. Vitamin C was also greater under each treatment, ranging from 35% to 45% (**Table 3**). Likewise, ‘Thurinus’ exposed to the 100:00, 75:25, and 50:50 B:R EOP treatments displayed 74%, 78%, and 78% greater concentrations of vitamin B_1_, respectively, compared to the control. ‘Thurinus’ developed a 46% higher concentration of vitamin B_3_ than the control when exposed to the 50:50 B:R EOP treatment. Similar to ‘Barlach’ and ‘Rouxai’, ‘Thurinus’ displayed increased concentrations of vitamin C under EOP treatments, ranging from 48% to 57% (**Table 3**).

**Table 3:** Influence of end-of-production (EOP) sole-source lighting providing a ratio (%) of 100:00, 75:25, and 50:50 blue:red (B:R) light or no EOP treatment (control) on vitamins B_1_, B_3_, B_6_, and C (µg·g^−1^ DM) of red leaf lettuce cultivars (*Lactuca sativa*) ‘Barlach’, ‘Rouxai’, and ‘Thurinus’.

EOP (B:R)	Vitamins			
	B_1_	B_3_	B_6_	C
‘Barlach’				
Control	1.2 b	1.7 a	40.8 a	331.9 b
100:00	2.8 ab	1.5 a	32.7 a	564.4 a
75:25	3.2 ab	1.4 a	29.2 a	557.1 a
50:50	3.8 a	1.1 a	25.5 a	584.5 a
‘Rouxai’				
Control	1.1 a	0.6 b	25.7 a	356.0 b
100:00	1.9 a	1.5 a	17.3 a	547.4 a
75:25	2.6 a	1.7 a	30.2 a	641.9 a
50:50	2.4 a	1.5 a	29.0 a	613.4 a
‘Thurinus’				
Control	0.8 b	1.2 b	39.8 a	298.0 b
100:00	3.1 a	2.0 ab	53.1 a	699.7 a
75:25	3.7 a	2.6 ab	41.0 a	636.2 a
50:50	3.7 a	3.5 a	31.9 a	577.1 a

Data represent the mean of three replications and 13 samples per cultivar. Different letters within columns indicate significantly different means according to Tukey’s honestly significant difference test (p < 0.05).

### 3.5 Anthocyanin and carotenoids

**Figure 4:**
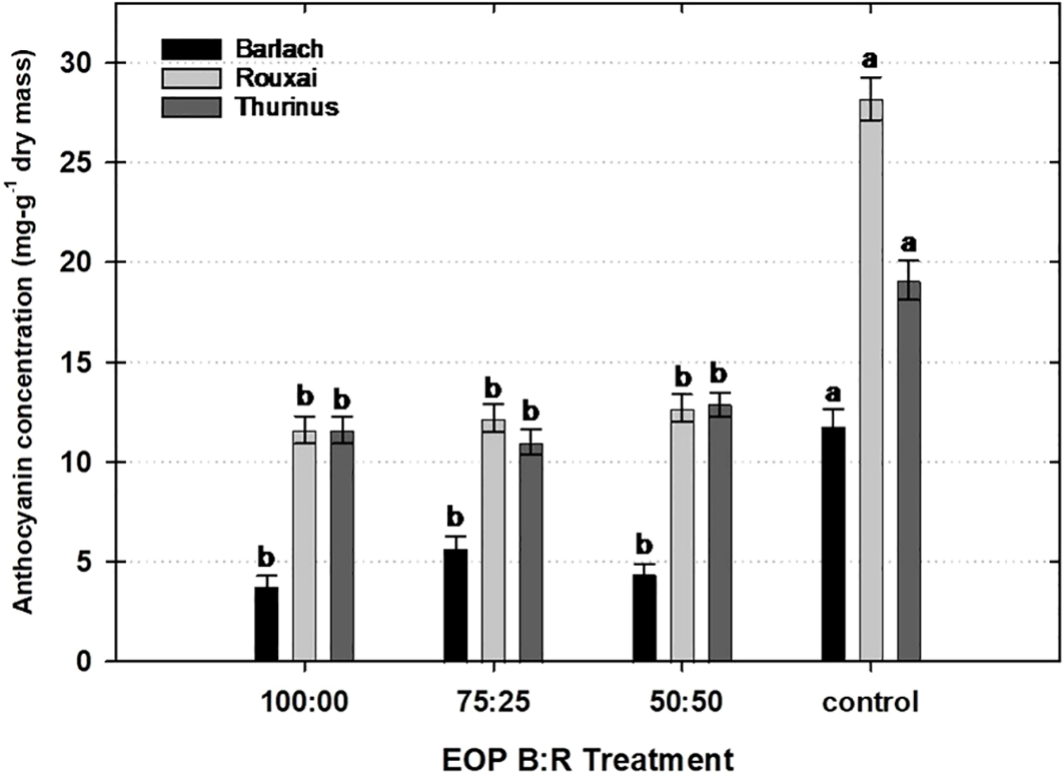
Influence of end-of-production (EOP) sole-source lighting providing a ratio (%) of 100:00, 75:25, and 50:50 blue:red (B:R) light or no EOP treatment (control) on anthocyanin concentration (mg·g^−1^ dry mass) of red leaf lettuce (*Lactuca sativa*) ‘Barlach’, ‘Rouxai’, and ‘Thurinus’. Different letters within each cultivar are significantly different based on Tukey’s honestly significant difference test (p < 0.05). Error bars represent the standard error of the mean.

Plants placed under EOP sole-source lighting had lower concentrations of anthocyanins than those under the control (**Figure 4**). ‘Barlach’ contained 68%, 52%, and 63% lower anthocyanin concentrations under the 100:00, 75:25, and 50:50 B:R EOP treatments, respectively, compared to the control. Likewise, ‘Rouxai’ placed under the 100:00, 75:25, and 50:50 B:R EOP treatments had 59%, 57%, and 55% lower anthocyanin concentrations than the control, respectively. Similarly, ‘Thurinus’ possessed 39%, 43%, and 33% lower anthocyanin concentrations than the control when placed under the 100:00, 75:25, and 50:50 B:R EOP treatments, respectively.

EOP treatments also altered the carotenoid composition and total carotenoid concentration of ‘Rouxai’ and ‘Thurinus’, but only the carotenoid composition of ‘Barlach’ (**Table 4**). The violaxanthin, neoxanthin, and α-carotene concentrations of untreated ‘Barlach’ were 37% to 62%, 64% to 73%, and 90% to 96% higher than those of plants exposed to EOP treatments, respectively. However, ‘Barlach’ under the 100:00, 75:25, and 50:50 B:R treatments had 62%, 60%, and 60% greater concentrations of β-carotene and 62%, 54%, and 52% higher concentrations of lutein, respectively, compared to the control. Similarly, untreated ‘Rouxai’ possessed between 10% and 50%, 56% and 75%, 66% and 78%, and 74% and 93% more violaxanthin, neoxanthin, zeaxanthin, and α-carotene than the plants exposed to EOP treatments. However, ‘Rouxai’ exposed to the 100:00 B:R EOP treatment had 31%, 38%, and 46% greater lutein concentrations than the control, 75:25, and 50:50 B:R treatments, respectively. Additionally, plants under the 100:00 B:R EOP treatments had 75%, 33%, and 29% higher β-carotene concentrations than the control, 75:25, and 50:50 B:R EOP treatments, respectively. ‘Rouxai’ under the 100:00 B:R EOP treatment possessed a 44% greater total carotenoid concentration than under both the 75:25 B:R and 50:50 B:R EOP treatments (**Table 4**). ‘Thurinus’ that was not exposed to an EOP treatment accumulated 42% more violaxanthin than plants under 50:50 B:R. Additionally, untreated ‘Thurinus’ possessed 53% to 68% more neoxanthin and 69% to 77% more zeaxanthin than plants exposed to EOP treatments. However, ‘Thurinus’ exposed to 100:00 B:R accumulated 38% and 72% greater lutein and β-carotene, respectively, compared to the control. The total carotenoid concentration of ‘Thurinus’ under the 100:00 B:R EOP treatments was 28% and 30% greater than the 75:25 and 50:50 B:R treatments, respectively (**Table 4**).

**Table 4:** Influence of end-of-production (EOP) sole-source lighting providing a ratio (%) of 100:00, 75:25, and 50:50 blue:red (B:R) light or no EOP treatment (control) on the violaxanthin, neoxanthin, zeaxanthin, lutein, α-carotene, β-carotene, and total carotenoid content (mg·g^−1^ dry mass) of red leaf lettuce cultivars (*Lactuca sativa*) ‘Barlach’, ‘Rouxai’, and ‘Thurinus’.

EOP (B:R)	Violaxanthin	Neoxanthin	Zeaxanthin	Lutein	α-Carotene	β-Carotene	Total carotenoids
‘Barlach’							
Control	0.8 a	1.5 a	0.6 ab	1.3 b	0.107 a	0.6 b	4.9 a
100:00	0.5 ab	0.4 b	0.8 a	3.4 a	0.011 b	1.7 a	6.9 a
75:25	0.4 b	0.3 b	0.5 b	2.8 a	0.004 b	1.5 a	5.7 a
50:50	0.3 b	0.3 b	0.4 b	2.7 a	0.005 b	1.5 a	5.4 a
‘Rouxai’							
Control	1.0 a	1.6 a	0.9 a	0.9 ab	0.101 a	0.6 c	4.8 ab
100:00	0.9 a	0.7 b	0.3 b	1.3 a	0.026 b	2.4 a	5.6 a
75:25	0.6 b	0.5 c	0.2 b	0.8 b	0.012 bc	1.6 b	3.7 b
50:50	0.5 b	0.4 c	0.2 b	0.7 b	0.007 c	1.7 b	3.6 b
‘Thurinus’							
Control	1.2 a	1.9 a	1.3 a	0.8 b	0.112 a	0.6 c	5.9 ab
100:00	1.1 a	0.9 b	0.4 b	1.4 a	0.021 b	2.7 a	6.4 a
75:25	0.9 ab	0.5 c	0.3 b	0.9 b	0.017 b	2.0 b	4.6 b
50:50	0.7 b	0.6 c	0.3 b	1.0 b	0.016 b	1.9 b	4.5 b

Data represent the mean of three replications and 13 samples per cultivar. Different letters within columns indicate significantly different means according to Tukey’s honestly significant difference test (p < 0.05).

## 4 Discussion

This study was designed to evaluate whether a 50% reduction in light intensity, enriched with B light, at EOP, could provide outcomes similar to or better than those of a higher-intensity white-light environment for plant morphology, nutrition, and leaf coloration. Due to space constraints, a low-intensity white light environment was not included. Therefore, direct intensity *vs*. spectrum comparisons, such as which had a greater effect on an observed result, are not possible, given that both light intensity and light quality differ between the control and the treatments. That said, halving the PPFD likely had a significant impact on crop growth. For example, the reductions in the SFM and SDM of ‘Rouxai’ and ‘Thurinus’ under EOP sole-source lighting can be attributed to the 6 days of reduced light intensity (**Table 2**). These results are congruent with outcomes from other studies, such as one completed by Zhou et al. (2022) in which growing romaine lettuce at PPFDs of 100 and 200 µmol·m^–2^·s^–1^ resulted in lower fresh mass than plants grown under a PPFD of 350, 500, or 600 µmol·m^–2^·s^–1^ and MDT of either 20°C or 30°C. Likewise, Kelly et al. (2020) reported that the SFM and SDM of ‘Rouxai’ were 51% and 31% greater when grown at a PPFD of 270 µmol·m^–2^·s^–1^ compared to a PPFD of 150 µmol·m^–2^·s^–1^. Surprisingly, the SFM and SDM of ‘Barlach’ were not affected by the combined reduction in light intensity and change in light quality, even though it was under EOP treatments for a longer duration than ‘Rouxai’ and ‘Thurinus’ (**Table 2**). This could indicate that ‘Barlach’ has a lower light saturation point than the other cultivars. Another possibility is that the growth rate of ‘Barlach’ plateaued between days 30 and 38. This would align with findings from Choi et al. (2003), in which the SFM and relative growth rate of butterhead lettuce ‘Omega’ were the greatest at 30°C/25°C, compared to 20°C/15°C, during the first 25 days, but by 35 days, there was no longer a difference.

For ‘Barlach’ and ‘Thurinus’, L* increased similarly across all EOP treatments when compared to the control, indicating that light quality at 150 µmol·m^–2^·s^–1^ PPFD did not impact foliage color. However, the higher light intensity supplied in the control treatment resulted in a darker foliage color than any of the lower intensity EOP treatments (**Figure 1**). The increase in L*, or leaf “lightness”, can be associated with a decrease in anthocyanins. This result aligns with the findings of Foster et al. (2018), who reported that the foliage of red leaf lettuce ‘Outredgeous’ grown under a PPFD of 500 µmol·m^–2^·s^–1^ was darker and more red than plants under a PPFD of 250 µmol·m^–2^·s^–1^. Furthermore, research by Owen et al. (2015) reported that EOP supplemental lighting providing 100 µmol·m^–2^·s^–1^ of a ratio (%) of 50:50 B:R light was more effective at decreasing L* and increasing a* of red leaf lettuce varieties ‘Magenta’, ‘Ruby Sky’, and ‘Cherokee’ than supplemental lighting providing 100 µmol·m^–2^·s^–1^ of 100:00 and 00:100 B:R light or 25 or 50 µmol·m^–2^·s^–1^ of 50:50 B:R light. This collectively indicates that higher light intensities, regardless of spectrum, affect anthocyanin synthesis and accumulation.

For ‘Rouxai’ and ‘Thurinus’, the concentration of some macro- and micronutrients increased under EOP sole-source lighting, relative to the control, while others decreased (**Figures 2**, **3**). Compared with other studies, it was unexpected that macro- and micronutrient concentrations generally increased under both reduced light intensity and increased B light percentage (Kopsell and Sams, 2013; Gerovac et al., 2016). A possible explanation for this difference is that the current study only provided B:R EOP sole-source lighting during the final 6 days of production, and the growth rate of ‘Rouxai’ may have plateaued by this point, limiting the effect on elemental composition of the entire plant (Choi et al., 2003). In contrast, ‘Thurinus’ placed under EOP for 8 days generally possessed greater concentrations of N, P, Ca, Mg, S, B, Cu, Mn, Mo, and Zn than the control (**Figures 2**, **3**). This was not surprising, as macro- and micronutrient concentrations generally increase under reduced light intensities due to nutrient dilution under higher light intensities and supplemented CO_2_ concentrations (Gerovac et al., 2016; Kuehny et al., 1991). However, there were differences between EOP treatments, indicating that, in addition to light intensity, light quality also influenced element accumulation. Additionally, there were no differences in the mineral nutrient concentration of ‘Barlach’, which, in combination with the lack of change in SFM or SDM, suggests the influence of cultivar-specific growth rates.

EOP lighting treatments generally increased WSV concentrations in all three cultivars. Plants exposed to EOP treatments showed increased vitamin C levels, compared to the control, but no differences within EOP treatments, suggesting that decreased light intensity rather than altered light quality was responsible (**Table 3**). However, Camejo et al. (2020) reported that lettuce ‘B. Diablotin’ accumulated more vitamin C when grown under a PPFD of 400 µmol·m^–2^·s^–1^ as opposed to a PPFD of 250 µmol·m^–2^·s^–1^. This could indicate cultivar-specific responses, in which reduced light intensity during less active growth phases stimulated vitamin C synthesis or decreased vitamin C utilization during the EOP phase. The synthesis of vitamin B_1_ can signal plant stress, with levels decreasing as the duration of the stress increases (Goyer, 2010). Interestingly, all three cultivars showed higher vitamin B_1_ concentrations under EOP sole-source lighting than under the control (numerically, although not always statistically), suggesting that the change in light intensity was a stress response. However, chlorophyll fluorescence (F_v_/F_m_) values did not indicate a reduction in maximum photosystem II efficiency. Hytönen et al. (2018) compared the vitamin B_9_ concentration of lettuce ‘Frillice’ grown under several spectra provided by LEDs alongside high-pressure sodium lighting and found that vitamin B_9_ was unaffected. Our study produced similar results for vitamins B_1_, B_3_, B_6_, and C, with no significant difference among EOP lighting treatments within cultivars. However, plants under EOP lighting treatments sometimes had higher vitamin B concentrations, supporting our hypothesis that light intensity may also affect B vitamin synthesis and accumulation.

Given that plants may synthesize anthocyanins to counteract oxidative damage, synthesis may be induced by high light intensities, specific wavelengths of PAR, and UV radiation (Lev-Yadun and Gould, 2008; Pollastri and Tattini, 2011). Previous studies have shown an increase in red lettuce leaf coloration when providing B light during production (Li et al., 2021) or supplemental B light during EOP (Kelly and Runkle, 2023; Zhu et al., 2024). Adjusting the light spectra to increase the amount (µmol·m^–2^·s^–1^) or fraction (%) of B light at EOP may be a viable strategy to increase anthocyanin concentration without the downside of inhibited leaf expansion (Son and Oh, 2013). Our approach differed in that we wanted to see whether a decrease in light intensity, coupled with an increase in the B fraction, could enhance leaf coloration. If so, it could be a potential strategy to reduce energy use at EOP. Contrary to previous studies, we observed no differences between the EOP B:R ratios used in the three cultivars. This indicates that at lower light intensities, altering light quality within PAR was ineffective at promoting anthocyanin synthesis. Also, all EOP B:R treatments had lower anthocyanin concentrations than the control (**Figure 4**), suggesting light intensity may have a greater effect on anthocyanin synthesis than light quality at PPFDs used in sole-source environments.

In ‘Rouxai’, total carotenoid concentration was 37% higher under the 100:00 B:R EOP treatment than under the 50:50 B:R treatment, but similar to the control. The greatest total carotenoid concentration in ‘Thurinus’ occurred in the 100:00 B:R EOP treatment, although none of the EOP treatments differed from the control. However, ‘Barlach’ displayed no difference in total carotenoid concentration (**Table 4**). Carotenoids are plant pigments that function as both an antenna for harvesting B light and protection against photooxidative damage via thermal dissipation (Stange and Flores, 2012; Jahns and Holzwarth, 2012). Furthermore, a recent study by Givens et al. (2023) found that β-carotene, lutein, neoxanthin, violaxanthin, and total carotenoid concentration of lettuce ‘Rex’ at harvest generally decreased as light intensity during seedling production increased from 60 to 600 μmol·m^−2^·s^−1^ PPFD. However, we did not see an increase in total carotenoid concentration at the reduced EOP light intensity, compared to the control. Increasing B light to 100:00 increased total carotenoid concentration relative to the 75:25 and 50:50 B:R EOP treatments. However, in all three cultivars, the levels of α-carotene, zeaxanthin, and neoxanthin generally were elevated in untreated plants exposed to higher light intensities. These differences again indicate cultivar-specific responses and may signal that, in addition to total carotenoid concentration, carotenoid composition alters with changes in light intensity and B light fraction.

In conclusion, reducing the EOP light intensity by half and increasing the B light fraction increased mineral nutrient concentrations and decreased biomass in ‘Rouxai’ and ‘Thurinus’; they also produced lighter foliage in ‘Barlach’ and ‘Thurinus’ and decreased anthocyanin concentration across cultivars. However, EOP monochromatic B light showed potential for increasing the total carotenoid concentration of ‘Rouxai’ and ‘Thurinus’. Transferring red leaf lettuce to a moderate intensity B-enriched EOP PPFD, with half the intensity of white light, did not enhance leaf redness. When promoting anthocyanin production under low-light conditions, particularly in indoor farms, EOP monochromatic B light becomes less necessary when high-intensity supplemental background light is provided. Therefore, practical approaches for growers to enhance red leaf lettuce coloration include EOP lighting, especially with additional B, or reducing production temperatures to promote anthocyanin production.

## Data Availability

The raw data supporting the conclusions of this article will be made available by the authors, without undue reservation.
